# Usefulness of waist-to-height ratio in screening incident hypertension among Japanese community-dwelling middle-aged and elderly individuals

**DOI:** 10.1186/s40885-020-00142-2

**Published:** 2020-05-15

**Authors:** Ryuichi Kawamoto, Asuka Kikuchi, Taichi Akase, Daisuke Ninomiya, Teru Kumagi

**Affiliations:** 1 Department of Community Medicine, Ehime University Graduate School of Medicine, Shitsukawa, Toon-city, Japan; 2 Department of Internal Medicine, Seiyo Municipal Nomura Hospital, 9-53 Nomura, Nomura-cho, Seiyo-City, Japan

**Keywords:** Waist-to-height ratio, Hypertension, Anthropometric indices, Community-dwelling individuals

## Abstract

**Background:**

The incidence of hypertension is increasing worldwide and obesity is one of the most significant risk factors. Obesity can be defined by various anthropometric indices such as body mass index (BMI), waist-to-hip ratio (WHpR), and waist-to-height ratio (WHtR). This study examined a range of anthropometric indices and their relationships with hypertension.

**Methods:**

This study included 768 men aged 70 ± 10 years and 959 women aged 70 ± 8 years from a rural village. The relationship between anthropometric indices (BMI, WHpR, and WHtR) and hypertension was examined using cross-sectional (baseline, *N* = 1727) and cohort data (follow-up, *N* = 419). Receiver operating characteristic (ROC) analysis was used to determine the predictive ability of obesity indices for hypertension in both genders. Logistic regression models were used to evaluate WHtR as a significant predictor of hypertension.

**Results:**

In the cross-sectional study, WHtR, BMI, and WHpR showed significant predictive abilities for hypertension in both genders, with WHtR showing the strongest predictive ability. Also, in the cohort study, WHtR showed a significant predictive ability for incident hypertension in both genders, and, for women, BMI as well as WHtR had also predictive ability. In the cross-sectional study, the optimal WHtR cutoff values were 0.53 (sensitivity, 44.3%; specificity, 80.2%) for men and 0.54 (sensitivity, 60.9%; specificity, 68.6%) for women. In the cohort study, the optimal WHtR values were 0.47 (sensitivity, 85.4%; specificity, 39.8%) for men and 0.51 (sensitivity, 66.7%; specificity, 58.2%) for women.

**Conclusions:**

The results suggest that WHtR is a useful screening tool for hypertension among Japanese middle-aged and elderly community-dwelling individuals.

## Background

The incidence of hypertension is increasing worldwide with the continuous increase in obesity prevalence [1]. Since obesity increases the risk of hypertension, addressing the obesity and hypertension epidemic is crucial [2]. Obesity can be defined by various obesity-related anthropometric indices such as body mass index (BMI), waist circumference (WC), waist-to-height ratio (WHtR), and waist-to-hip ratio (WHpR). The World Health Organization recommends the use of some anthropometric parameters as screening markers for individuals at risk for cardiovascular disease (CVD) as they can be determined easily and inexpensively [3] Epidemiological studies have shown that these anthropometric indices predict incident hypertension [4–9]. BMI is the most widely used indicator of obesity, but it does not reflect central fat distribution [10, 11]. In Japan, cross-sectional and prospective studies have demonstrated a strong association between WHtR and hypertension [4, 5, 12]. However, which index best predicts the development of hypertension remains controversial [13–16], and more specifically, there is a lack of consensus on the best predictive indicator of hypertension among Japanese middle-aged and elderly individuals.

To address this controversy, we investigated the relationship between baseline visceral obesity indices (BMI, WHpR, and WHtR) and potential risk factors and hypertension using cross-sectional and prospective cohort data from community-dwelling middle-aged and elderly individuals.

## Methods

### Study participants and data collection

This study enrolled a population-based sample of community-dwelling Japanese adults from the Nomura Health and Welfare Center in a rural town in the Ehime prefecture of Japan. Factors for increased CVD risk were examined from annual health checkup data [17]. Follow-up assessment cycles are performed every 3 years.

**Fig. 1:**
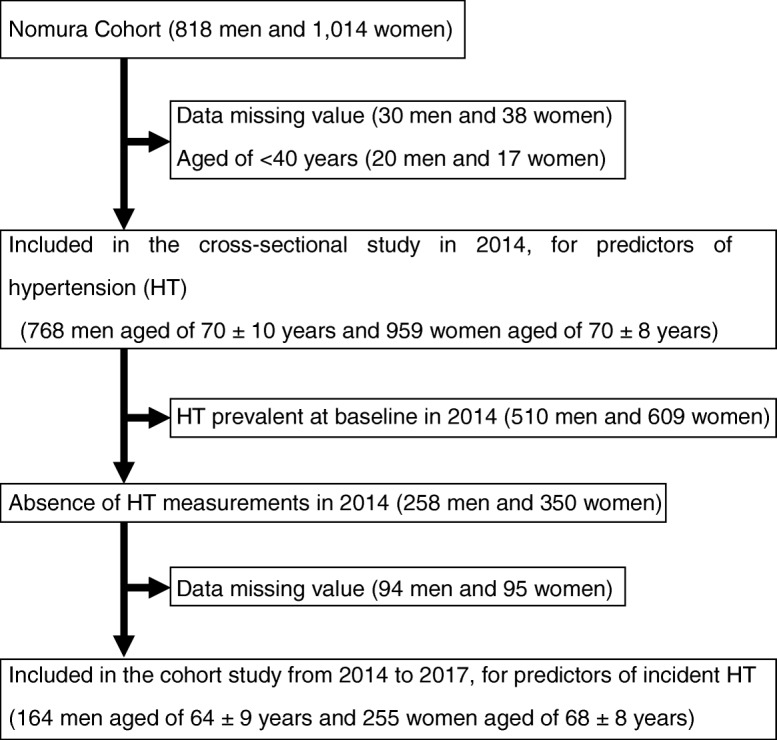
Flowchart. For the cross-sectional analyses, data from the 2014 cycle (*n* = 1727) were used. For the longitudinal analyses, only participants in whom hypertension was not prevalent at baseline in 2014 were included (*n* = 419)

Overall, 1832 community-dwelling participants aged 20–95 years (818 men and 1014 women) were enrolled between April and November 2014. In the present study, the analysis was restricted to participants aged ≥40 years without any missing baseline data. The initial dataset consisted of 1727 participants (768 men and 959 women) aged 40–95 years, on whom the follow-up survey, spanning 3 years, was performed. Of these participants, 1308 were excluded due to the presence of hypertension (*N* = 1119) and missing data (*N* = 189), especially data on WC and blood pressure. The final dataset comprised 419 non-hypertensive participants (164 men and 255 women). Figure 1 shows a flowchart describing the inclusion of participants.

All participants provided written informed consent, and the Nomura study was approved by the Ehime University Medical School Ethics Committee. All procedures performed in the study involving human participants were in accordance with the ethical standards of the institutional research committee in which the study was conducted (IRB Approval number: 1402009).

### Anthropometric and laboratory measurements

Baseline anthropometric indices such as BMI, WC, WHpR, and WHtR were measured. BMI was calculated by dividing weight in kilograms (kg) by height in meters squared (m^2^). WC was measured in the horizontal plane at the mid-point between the anterior iliac crest and the inferior margin of the rib. WHtR was calculated as WC (cm)/height (cm). WHpR was calculated as WC (cm)/hip circumference (cm). In addition, lifestyle-related factors such as smoking, alcohol consumption, and regular exercise habits were also investigated by individual interviews conducted using a structured questionnaire. Smoking habits were determined by multiplying the number of years the person has smoked by the average number of packs smoked per day (pack year), and participants were classified as never smokers, former smokers, light smokers (< 30 pack year), and heavy smokers (≥30 pack year) [18]. Alcohol consumption was measured using the traditional Japanese unit of alcohol, go, which is equivalent to 22.9 g of ethanol, and the participants were classified as non-drinkers, occasional drinkers (< 1 unit/day), daily light drinkers (< 2 unit /day), and daily heavy drinkers (≥2 unit/day) [19]. Systolic blood pressure (SBP) and diastolic blood pressure (DBP) were measured twice using an automatic oscillometric blood pressure recorder, on the right upper arm of the subjects with an appropriate-sized cuff in the sedentary position after having rested for at least 5 min. The two values were then averaged.

For all these individuals, triglycerides (TG), high-density lipoprotein cholesterol (HDL-C), low-density lipoprotein cholesterol (LDL-C), hemoglobin A1c (HbA1c), serum uric acid (SUA), and creatinine (Cr) were measured during an overnight fast of over 11 h. The estimated glomerular filtration ratio (eGFR) was calculated using the Chronic Kidney Disease Epidemiology Collaboration (CKD-EPI) equations modified by the Japanese coefficient (eGFR_CKDEPI_): Male, Cr ≤0.9 mg/dl, 141 × (Cr/0.9) ^–0.411^ × 0.993 ^age^ × 0.813; Cr > 0.9 mg/dl, 141 × (Cr/0.9) ^–1.209^ × 0.993 ^age^ × 0.813; Female, Cr ≤0.7 mg/dl, 144 × (Cr/0.7) ^–0.329^ × 0.993 ^age^ × 0.813; Cr > 0.7 mg/dl, 144 × (Cr/0.7) ^–1.209^ × 0.993 ^age^ × 0.813 [20].

### Criteria for clinical diagnosis of hypertension

Normotension was defined as not being on antihypertensive medication and having a SBP < 120 mmHg and DBP < 80 mmHg. Prehypertension was defined as not being on antihypertensive medication and having a SBP of 120 to 139 mmHg and/or DBP 80 to 89 mmHg. Hypertension was defined as being on antihypertensive medication and/or having SBP ≥140 mmHg and/or DBP ≥90 mmHg according to the definitions of the Joint National Committee 7 [21].

### Statistics

All the data were analyzed using the IBM Statistical Package for Social Sciences version 21.0 (IBM SPSS Statistics Inc. Chicago IL. USA). Continuous variables were presented as means ± standard deviation (SD) if the data were normally distributed and as medians (interquartile ranges) if the distributions were skewed (e.g., TG and HbA1c). Subjects were divided into two groups based on gender, **and** differences among the groups were analyzed by Student’s t-test for continuous variables or a chi-squared (χ^2^) test for categorical variables. For all analyses, parameters with non-normal distributions were used after log-transformation. Multiple logistic regression analysis was used to evaluate the contribution of the baseline WHtR and confounding factors (i.e., gender, age, smoking status, drinking status, exercise habits, presence of CVD, TG, HDL-C, LDL-C, use of antidyslipidemic medication, HbA1c, use of antidiabetic medication, eGFR, and SUA) on the prevalence of hypertension in the cross-sectional study and on the incidence of hypertension in the cohort study. If any independent variables are correlated with each other (*r* ≥ 0.6) (known as multicollinearity) the variable was removed from the multivariate analysis (e.g., BMI, WHtR, and WHpR). In addition, areas under the receiver operating characteristic (ROC) curves were determined for each variable to identify the predictors of hypertension. A ROC curve is a plot of sensitivity (true positive) versus 1–specificity (false positive) for different cutoff points of a parameter. Area under the ROC curve (AUC) is a summary of the overall diagnostic accuracy of the test including standard errors. Predictive values were calculated as sensitivity/[sensitivity+(1-specificity)] (positive predictive value) and specificity/[(1-sensitivity) + specificity] (negative predictive value). The optimal cutoff values were defined as the point at which the value of sensitivity + specificity − 1 was maximum (Youden’s index) [22]. A *p*-value < 0.05 was considered significant.

## Results

### Baseline characteristics of study subjects categorized by presence of hypertension in the cross-sectional and cohort studies

A total of 1727 participants were included at baseline in this cross-sectional study. Baseline characteristics of the subjects categorized by hypertension status are shown in Table 1. The study included 768 men aged 70 ± 10 (range, 40–95) years and 959 women aged 70 ± 8 (range, 41–90) years. The proportion of men was 42.4% in the normotension group and 45.6% in the hypertension group. Age, BMI, WC, WHpR, WHtR, smoking habit, prevalence of CVD, SBP, DBP, use of antihypertensive medication, TG, use of antidyslipidemic medication, HbA1c, use of antidiabetic medication, and SUA were significantly higher in the hypertension group, but HDL-C, LDL-C, and eGFR were significantly lower. There were no differences in gender, drinking status, or exercise habits. In the cohort study as shown in Table 2, the proportion of men was 38.0% in the normotension group and 43.2% in the hypertension group. Age, BMI, WC, WHtR WHpR, SBP, DBP, and SUA were significantly higher in the hypertension group, but prevalence of exercise habits was significantly lower.

**Table 1:** Baseline characteristics of subjects according to hypertension status in the cross-sectional study

		Normotension	Hypertension	***P***-value*
Baseline Characteristics	***N*** = 1727	***N*** = 608	***N*** = 1119	
Gender (male, %)		258 (42.4)	510 (45.6)	0.224
Age (years)		66 ± 9	72 ± 8	**< 0.001**
BMI (kg/m^2^)		21.8 ± 2.8	23.4 ± 3.2	**< 0.001**
Waist circumference (cm)		78.9 ± 8.0	82.7 ± 8.9	**< 0.001**
WHtR		0.51 ± 0.05	0.54 ± 0.06	**< 0.001**
WHpR		0.88 ± 0.06	0.91 ± 0.06	**< 0.001**
Smoking habit (never/past/light/heavy (%))		71.2/15.5/5.3/8.1	72.4/20.5/1.7/5.5	**< 0.001**
Drinking status (never/occasional/light/heavy (%))		50.5/25.2/8.7/15.6	50.3/20.6/10.3/18.9	0.069
Exercise habits (%)		36.0	38.0	0.435
Cardiovascular disease (%)		4.6	8.0	**0.009**
Systolic blood pressure (mmHg)		122 ± 12	144 ± 15	**< 0.001**
Diastolic blood pressure (mmHg)		72 ± 8	81 ± 10	**< 0.001**
Antihypertensive medication (%)		0	68.7	**< 0.001**
Triglycerides (mg/dl)		83 (61–114)	91 (69–129)	**< 0.001**
HDL cholesterol (mg/dl)		68 ± 18	64 ± 16	**< 0.001**
LDL cholesterol (mg/dl)		122 ± 29	119 ± 30	**0.047**
Antidyslipidemic medication (%)		14.3	26.5	**< 0.001**
Hemoglobin A1c (%)		5.6 (5.4–5.8)	5.7 (5.5–6.0)	**< 0.001**
Antidiabetic medication (%)		5.6	10.5	**< 0.001**
Serum uric acid (mg/dL)		5.1 ± 1.3	5.4 ± 1.4	**< 0.001**
Estimated GFR (ml/min/1.73 m^2^/year)		74.9 ± 9.8	69.6 ± 12.2	**< 0.001**

*BMI* body mass index; *WHtR* waist/height ratio; *WHpR* waist/hip ratio; *HDL* high-density lipoprotein; *LDL* low-density lipoprotein; *GFR* glomerular filtration ratio. Data presented are mean ± standard deviation. Data for triglycerides and hemoglobin A1c is skewed, and presented as median (interquartile range) values. * *P*-value: Student’s t-test for the continuous variables or the χ^2^ test for the categorical variables. Bold values indicate significance (*p* < 0.05)

**Table 2:** Baseline characteristics of subjects according to hypertension status in the cohort study

		Normotension	Hypertension	***P***-value*
Baseline Characteristics	***N*** = 419	***N*** = 324	***N*** = 95	
Gender (male, %)		123 (38.0)	41 (43.2)	0.403
Age (years)		65 ± 9	67 ± 9	**0.036**
BMI (kg/m^2^)		21.4 ± 2.6	22.5 ± 2.7	**< 0.001**
Waist circumference (cm)		78.0 ± 7.7	80.5 ± 7.5	**0.005**
WHtR		0.50 ± 0.05	0.52 ± 0.06	**0.001**
WHpR		0.87 ± 0.06	0.89 ± 0.06	**0.017**
Smoking habit (never/past/light/heavy (%))		71.9/15.1/4.6/8.3	74.7/12.6/5.3/7.4	0.910
Drinking status (never/occasional/light/heavy (%))		51.5/25.3/7.1/16.0	43.2/25.3/10.5/21.1	0.361
Exercise habits (%)		38.3	26.3	**0.038**
Cardiovascular disease (%)		3.1	3.2	1.000
Systolic blood pressure (mmHg)		119 ± 11	130 ± 8	**< 0.001**
Diastolic blood pressure (mmHg)		71 ± 8	77 ± 7	**< 0.001**
Antihypertensive medication (%)		0	0	1.000
Triglycerides (mg/dl)		81 (59–109)	88 (63–126)	0.285
HDL cholesterol (mg/dl)		69 ± 17	67 ± 19	0.498
LDL cholesterol (mg/dl)		123 ± 29	120 ± 28	0.390
Antidyslipidemic medication (%)		16.0	15.8	1.000
Hemoglobin A1c (%)		5.6 (5.4–5.8)	5.6 (5.4–5.8)	0.494
Antidiabetic medication (%)		4.6	3.2	0.774
Serum uric acid (mg/dL)		5.0 ± 1.3	5.4 ± 1.3	**0.002**
Estimated GFR (ml/min/1.73 m^2^/year)		76.0 ± 10.0	75.0 ± 8.0	0.361

Data presented are mean ± standard deviation. Data for triglycerides and hemoglobin A1c is skewed, and presented as median (interquartile range) values. * *P*-value: Student’s t-test for the continuous variables or the χ^2^ test for the categorical variables. Bold values indicate significance (*p* < 0.05)

### Results of the ROC curve analyses to identify optimal obesity indices to distinguish subjects with hypertension in the cross-sectional and cohort studies

**Fig. 2:**
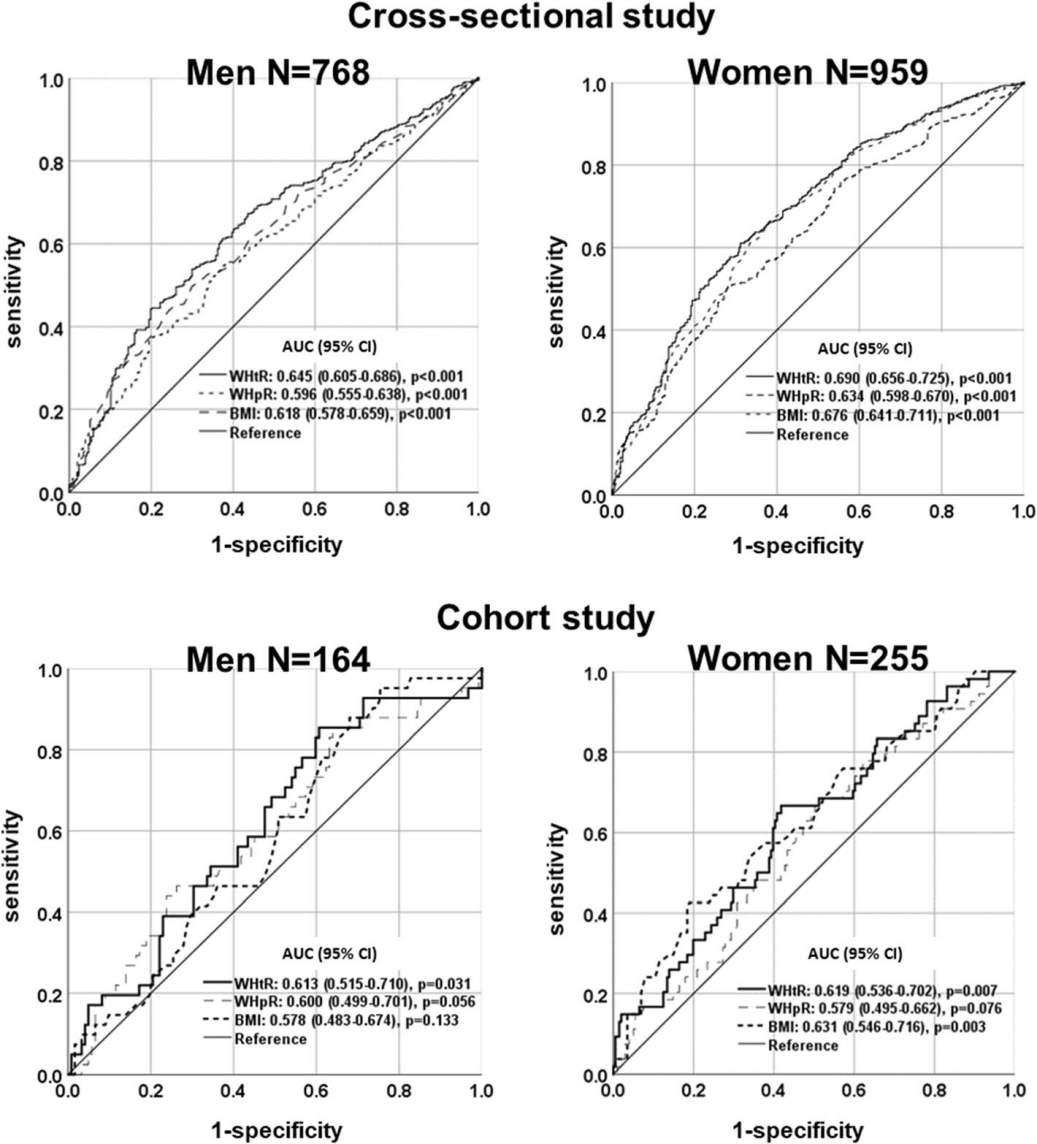
Area under the receiver operating curve (AUC) values (95% CI) for selected obesity measurements to distinguish subjects with hypertension in the cross-sectional and cohort studies

Figure 2 shows the AUC for BMI, WHpR, and WHtR for hypertension in both genders using ROC analyses. In the cross-sectional study, WHtR, BMI, and WHpR showed significant predictive ability for incident hypertension in both genders. Also, in the cohort study, WHtR showed a significant predictive ability for incident hypertension in both genders, and, for women, BMI as well as WHtR had also predictive ability. As shown in Table 3, in men WHtR showed significantly stronger predictive ability than WHpR and BMI, but in women WHtR as well as BMI were stronger than WHpR. In cohort study, there was no any difference of three devices.

**Table 3:** The difference among the AUC values of baseline indices of obesity in the cross-sectional and cohort studies.

Cross-sectional study	***N*** = 1727	AUC (95% CI)	***P***-value*
Men	*N* = 768		
WHtR - WHpR		0.049 (0.025–0.072)	**< 0.001**
WHtR - BMI		0.202 (0.003–0.050)	**0.027**
WHpR - BMI		−0.022 (−0.058–0.014)	0.228
Women	*N* = 959		
WHtR - WHpR		0.057 (0.036–0.077)	**< 0.001**
WHtR - BMI		0.014 (−0.008–0.036)	0.202
WHpR - BMI		−0.042 (− 0.077−0.008)	**0.016**
**Cohort study**	***N*** **= 419**	**AUC (95% CI)**	*P*-value*
Men	*N* = 164		
WHtR - WHpR		0.013 (−0.053–0.079)	0.702
WHtR - BMI		0.034 (−0.030–0.099)	0.297
WHpR - BMI		0.021 (−0.076–0.119)	0.667
Women	*N* = 255		
WHtR - WHpR		0.040 (−0.009–0.089)	0.107
WHtR - BMI		−0.012 (− 0.064–0.040)	0.648
WHpR - BMI		−0.052 (− 0.133–0.029)	0.206

*AUC* area under the curve. * *P*-value: ROC analysis. Bold values indicate significance (*p* < 0.05)

### Odds ratios and 95% confidence interval (CI) for hypertension by quartile of WHtR in the cross-sectional and cohort studies

To further investigate whether WHtR can explain hypertension independently of other confounding factors, a multiple logistic regression analysis was performed. Hypertension was the dependent variable and various confounding factors (e.g., age, smoking status, drinking status, exercise habits, presence of CVD, SBP, DBP, use of antihypertensive medication, TG, HDL-C, LDL-C, use of antidyslipidemic medication, SUA, and eGFR) were the explanatory variables (Table 4). In both the cross-sectional and cohort studies, increased WHtR showed an increasing trend with increased prevalence and incidence of hypertension.

**Table 4:** Odds ratios and 95% CI for hypertension of subjects according to quartiles of baseline WHtR in the cross-sectional and cohort studies

		Odds ratio (95% CI)				
		Waist-to-height ratio				
		Quartile 1	Quartile 2	Quartile 3	Quartile 4	
	Men	0.38–0.47	0.47–0.51	0.51–0.54	0.54–0.74	
	Women	0.36–0.49	0.49–0.54	0.54–0.58	0.58–0.80	
Cross-sectional study	***N*** = 1727	***N*** = 432	***N*** = 431	***N*** = 432	***N*** = 432	***P***-Value
Hypertension						
Incidence		202 (46.8%)	254 (58.9%)	312 (72.2%)	351 (81.3%)	**< 0.001**
Non-adjusted		1	**1.63 (1.25–2.14)**	**2.96 (2.23–3.93)**	**4.93 (3.63–6.71)**	**< 0.001**
Gender and age-adjusted		1	**1.55 (1.17–2.05)**	**2.71 (2.02–3.63)**	**4.13 (3.01–5.67)**	**< 0.001**
Multivariate-adjusted		1	**1.44 (1.07–1.94)**	**2.35 (1.71–3.22)**	**3.44 (2.43–4.88)**	**< 0.001**
**Cohort study**	***N*** **= 419**	***N*** **= 170**	***N*** **= 127**	***N*** **= 79**	***N*** **= 43**	***P*-Value**
Hypertension						
Incidence		27 (15.9%)	30 (23.6%)	24 (30.4%)	14 (32.6%)	**0.022**
Non-adjusted		1	1.64 (0.92–2.93)	**2.31 (1.23–4.35)**	**2.56 (1.20–5.46)**	**0.022**
Gender and age-adjusted		1	1.58 (0.88–2.83)	**2.13 (1.12–4.04)**	**2.28 (1.04–5.00)**	0.064
Multivariate-adjusted		1	1.54 (0.82–2.92)	**2.43 (1.18–5.03)**	**2.52 (1.03–6.20)**	0.074

*CI* confidence interval. *Multivariate-adjusted for gender, age, smoking status, drinking status, exercise habits,presence of cardiovascular disease, triglycerides, HDL-cholesterol, LDL-cholesterol, use of antidyslipidemic medication, HbA1c, use of antidiabetic medication, eGFR, and serum uric acid. Bold values indicate significance (*p* < 0.05)

### Best cutoff values of WHtR to predict hypertension in the cross-sectional and cohort studies

In the cross-sectional study, the optimal WHtR cutoff values for predicting hypertension were 0.53 (sensitivity, 44.3%; specificity, 80.2%) for men and 0.54 (sensitivity, 60.98%; specificity, 68.6%) for women (Table 5). In the cohort study, the optimal WHtR values were 0.47 (sensitivity, 85.4%; specificity, 39.8%) for men and 0.51 (sensitivity, 66.7%; specificity, 58.2%) for women.

**Table 5:** Best cutoff values of baseline WHtR to predict hypertension in the cross-sectional and cohort studies

Cross-sectional study	***N*** = 1727	Cutoff value	Sensitivity	Specificity	PPV	NPV	Efficiency
Men	*N* = 768	0.5250	44.3%	80.2%	69.1%	59.0%	62.2%
Women	*N* = 959	0.5376	60.9%	68.6%	66.0%	63.7%	64.8%
**Cohort study**	***N*** **= 419**	**Cutoff value**	**Sensitivity**	**Specificity**	**PPV**	**NPV**	**Efficiency**
Men	*N* = 164	0.4701	85.4%	39.8%	58.7%	73.2%	62.6%
Women	*N* = 255	0.5136	66.7%	58.2%	61.5%	62.6%	62.5%

*PPV* positive predictive value; *NPV* negative predictive value

## Discussion

In the present study, WHtR was significantly and independently associated with the prevalence of hypertension in this cross-sectional study and the incidence of hypertension in this cohort study. The mean AUC of the WHtR (men, AUC = 0.613; 95% CI, 0.515–0.710, *p* = 0.031; women, AUC = 0.619; 95% CI, 0.536–0.702, *p* = 0.007) was higher than that of the BMI (men, AUC = 0.578; 95% CI, 0.483–0.674, *p* = 0.133; women, AUC = 0.631; 95% CI, 0.546–0.716, *p* = 0.003) and WHpR (men, AUC = 0.600; 95% CI, 0.499–0.701, *p* = 0.056; women, AUC = 0.579; 95% CI, 0.495–0.662, *p* = 0.076) which are conventional obesity indices among both genders. To the best of our knowledge, few epidemiologic studies have quantified the relevance of WHtR in predicting incident hypertension in Japanese middle-aged and elderly community-dwelling individuals.

A variety of anthropometric indicators have been developed to identify CVD risk factors such as hypertension, diabetes, and dyslipidemia [23]. To date, however, no definitive measurement tool has been developed for the prediction of hypertension [7]. This study examined three different indicators of fat distribution—BMI, WHpR, and WHtR—which have reportedly been associated with the prevalence of hypertension in both genders. In previous cross-sectional studies, ROC curve analyses demonstrated that WHtR was a better indicator than other indices among young men and women, but BMI and WC were more sensitive markers among middle-aged men and women [16]. Meanwhile, for middle-aged and elderly Brazilians, Dutra et al. [8] reported that WC and WHpR had a stronger relationship with prevalence of hypertension compared to other indices. Population-based prospective studies among Japanese men and women showed that both BMI and WC were significant predictors for hypertension [4]. In meta-analyses involving 309,585 participants (men: 51.6 ± 9.6 years; women: 51.0 ± 9.3 years), WHtR had the strongest prediction ability for hypertension, which was also confirmed in subgroup analyses based on gender and country [9]. Also, in our Japanese middle-aged and elderly participants, an increasing WHtR was found to be a significant indicator for the incidence of hypertension, and in women high BMI also have the highest correlation with incident hypertension. This result may be due to the following two reasons. First, WHR can distinguish central obesity from lower body and general obesity and carries some information on both overall obesity and abdominal obesity, since WHtR is positively correlated with BMI and WC. Second, the explanation for this finding might be the difference in body composition by gender [24]. Men tend to have greater skeletal muscles than women and women tend to have a higher percentage of body fat than men [25]. Thus, the inconsistent association of obesity indices and hypertension may be due to different races, age groups, and sexes [16].

The mechanisms that lead to increased incidence of hypertension in individuals with increased WHtR remain to be elucidated. BMI is the most common anthropometric index and is strongly related to body fat, but is not necessarily related to abdominal obesity because BMI cannot distinguish between people with high muscle mass and those with excess fat. It is increasingly clear that BMI is a rather poor indicator of percent body fat, whereas WC, WHpR, and WHtR are used as surrogate markers for body fat centralization [5, 26]. WC accurately reflects the degree of visceral fat rather than the absolute degree of adiposity, but it does not take into account height differences and may overestimate or underestimate the risk of CVD [12, 27]. Hsieh et al. [12] showed that people with a noticeably large WC may have the same health risks as the above items regardless of their height, but short people have higher health risks than tall people in the moderately large WC population of Japanese men. Of these three measurements, only the WHpR considers differences in body structure and is an anthropometric measure commonly used to characterize regional adiposity.

WHtR is a simple and practical anthropometric index to identify higher metabolic risks in normal and overweight Japanese men and women [12, 28, 29]. Hsieh et al. [29] suggested that the optimal value for WHtR was 0.5 for risk factors defined by the American Heart Association, the National Heart, Lung, and Blood Institute, and the International Diabetes Federation and approximately 0.5 for other risk factors in both genders. For non-overweight Korean adults, a higher incidence of hypertension was observed in the WHtR ≥0.5 group than the WHtR < 0.5 group [7]. A Chinese study found that a WHtR cutoff value of ≥0.5 identified people with high adiposity and was strongly associated with hypertension [30]. In our study, a positive association between WHtR and the incidence of hypertension was observed in Japanese adults and a WHtR cutoff value of 0.47 to 0.53 may be a better predictor of incident hypertension.

There are several possible research limitations that could affect our study. First, the cross-sectional study design does not establish a cause-and-effect relationship between conventional obesity indices and the presence of hypertension. Second, we must consider the influence that medications for hypertension, dyslipidemia, and hyperglycemia had on the present findings. Third, the measurements of WHtR and confounding factors are based on a single evaluation, which may introduce a misclassification bias. Fourth, the longitudinal analyses were reflected by a relatively smaller sample size and discrepancies in the sequential measurements of confounders in 2014 and 2017. The cohort was slightly younger and healthier compared to participants not included in the longitudinal analyses, which may have caused an underestimation of incident hypertension at the three-year follow-up. Thus, the demographics and referral source may limit the generalizability of the obtained results.

## Conclusion

The findings of this present study suggest that WHtR is strongly associated with the prevalence and incidence of hypertension among Japanese community-dwelling individuals. Thus, WHtR might be an important marker for the assessment of risk and become a therapeutic target for hypertension. For healthy community residents, prospective population-based studies are necessary to investigate interventions such as effective lifestyle improvement and other interventions to control WHtR in adults.

## Data Availability

Not applicable.

## Abbreviations

BMI: Body mass index
WC: Waist circumference
WHtR: Waist-to-height ratio
WHpR: Waist-to-hip ratio
CVD: Cardiovascular disease
SBP: Systolic blood pressure
DBP: Diastolic blood pressure
TG: Triglycerides
HDL-C: High-density lipoprotein cholesterol
LDL-C: Low-density lipoprotein cholesterol
HbA1c: Hemoglobin A1c
SUA: Serum uric acid
Cr: Creatinine
eGFR: Estimated glomerular filtration ratio
CKD-EPI: Chronic Kidney Disease Epidemiology Collaboration
eGFR_CKDEPI_: Equations modified by the Japanese coefficient
SD: Standard deviation
ROC: Receiver operating characteristic
AUC: Area under the ROC curve
